# Elevated Serum Uric Acid Is Associated with Greater Bone Mineral Density and Skeletal Muscle Mass in Middle-Aged and Older Adults

**DOI:** 10.1371/journal.pone.0154692

**Published:** 2016-05-04

**Authors:** Xiao-wei Dong, Hui-yuan Tian, Juan He, Chen Wang, Rui Qiu, Yu-ming Chen

**Affiliations:** Guangdong Provincial Key Laboratory of Food, Nutrition and Health, School of Public Health, Sun Yat-sen University, Guangzhou 510080, People's Republic of China; IPK, GERMANY

## Abstract

**Background and objective:**

Previous studies have suggested a positive link between serum uric acid (UA) and bone mineral density (BMD). In this study, we re-examined the association between UA and BMD and further explored whether this was mediated by skeletal muscle mass in a general Chinese population.

**Method:**

This community-based cross-sectional study was conducted among 3079 (963 men and 2116 women) Chinese adults aged 40–75 years. Face-to-face interviews and laboratory analyses were performed to determine serum UA and various covariates. Dual-energy X-ray absorptiometry was used to assess the BMD and appendicular skeletal muscle mass. The skeletal muscle mass index (SMI = ASM/Height^2^, kg/m^2^) for the total limbs, arms, and legs was then calculated.

**Results:**

The serum UA was graded and, in general, was significantly and positively associated with the BMD and muscle mass, after adjustment for multiple covariates in the total sample. Compared with participants in lowest quartile of UA, those participants in highest quartile showed a 2.3%(whole body), 4.1%(lumbar spine), 2.4%(total hip), and 2.0% (femoral neck) greater BMDs. The mean SMIs in the highest (vs. lowest) quartile increased by 2.7% (total), 2.5% (arm), 2.7% (leg) respectively. In addition, path analysis suggested that the favorable association between UA and BMD might be mediated by increasing SMI.

**Conclusion:**

The elevated serum UA was associated with a higher BMD and a greater muscle mass in a middle-aged and elderly Chinese population and the UA-BMD association was partly mediated by muscle mass.

## Introduction

Osteoporosis and sarcopenia, the results of musculoskeletal aging, are a tremendous and growing public health problem for the aging society [1, 2]. Muscle and bone are inextricably linked, both functionally and mechanically. The muscle-derived mechanical loading stimulates bone development and maintenance [3, 4]. Moreover, biochemical interactions could exist between muscle and bone via soluble factors, in a paracrine and endocrine manner [5]. For example in vitro experiments and animal model observed that insulin-like growth factor-1 (IGF-1), whose one potential source is muscle, promotes osteoblast proliferation, survival, and differentiation [6–8], and is associated with the increased bone mass [8]. Considering the mechanism of interaction and the functionally synergistic effect of muscle and bone, new strategies and administrations to simultaneously address muscle and bone aging as a whole are being called on urgently [9, 10].

Oxidative stress plays a role in the pathogenesis of both muscle and bone aging [11]. *In vitro*, reactive oxygen species (ROS) are observed to suppress osteoblast generation and differentiation, to enhance osteoclast development and activity, and to promote myofibrillar proteolysis [12, 13]. Lower levels of plasma antioxidants are associated with a higher risk of poor muscle strength [14], osteoporosis [15, 16], bone loss, and fractures in humans [17, 18]. Uric acid (UA), a metabolite of purine, is considered to be a risk factor for various chronic diseases, such as gout and cardiovascular diseases [19, 20]. However, an increasing number of studies recognize that UA might function as a potent antioxidant, combating oxidative stress [21], and could play a role in the prevention and attenuation of some degenerative diseases, especially some neurological diseases and cancers [22–24]. Its role in musculoskeletal health has not yet been fully elucidated.

To date, several studies have investigated the relationship between UA and bone health. Cross-sectional studies showed that higher serum UA was associated with greater BMD or lower prevalence of osteoporosis among middle-aged and older adults both in Western and Asian populations [25–28], but not in an American general population [29]. In longitudinal studies, uric acid was inversely associated with the risk of fractures in Korean men [30] and bone loss in Australian women [31], but not with fractures in other two western studies [32, 33]. Moreover, one study even reported a U-shaped relationship between UA and fractures in men [33]. Therefore, the UA-bone association remained speculative; more studies in a variety of populations world-wide are needed before getting a conclusive result in future. In addition, up to date, little has been known regarding the UA-muscle relationship and whether serum UA exerts an indirect effect on bone metabolism by affecting muscle, although “it is time to consider ‘sarco-osteopenia’” [34].

In this cross-sectional study including Chinese middle-aged and older adults, we examined the association between the serum concentration of UA, and the BMD and skeletal muscle mass, and further explored whether the potential UA-BMD relationship is mediated by muscle mass in Chinese middle-aged and older adults.

## Material and Methods

### Study participants

The present study originated from on the first follow-up survey of the Guangzhou Nutrition and Health Study (GNHS), a community-based prospective cohort designed to identify the determinants of CVDs and bone health, as described in previous reports [35, 36]. Briefly, from July 2008 to June 2010, we enrolled 3,169 apprently “healthy” participants aged 40–75 years living in urban Guangzhou city for at least 5 years via invitation letters, local advertisements, and referrals. During April 2011- January 2013, 2523 participants were successfully followed up. After that time, we recruited an additional 877 participants to supplement our cohort using the same methods as we did at baseline during January—October 2013. Among 3400 participants who completed the survey during 2011–2013, we excluded 321 subjects due to the following conditions:1) chronic renal failure (n = 6); 2) liver cirrhosis (n = 3); 3) cancers (n = 32); 4) rheumatoid arthritis (n = 40); 5) gout (n = 110); 6) incompletion of assessments serum UA (n = 102) and BMDs (n = 28). Thus, 3079 participants were eventually included in this study. The study was approved by the Ethics Committee of the School of Public Health of Sun Yat-sen University. Each participant was given written informed consent at the baseline and at the follow-up.

### Data collection and measurements

#### General data collection

A face-to-face interview using structured questionnaires was used to collect general information on the demographics, socioeconomic status, age at menarche, and years since menopause (YSM), alcohol consumption, cigarette smoking, disease history, medications affecting bone metabolism (including hormone therapy, bisphosphonate, calcitonin, vitamin D, etc.), and use of calcium and multivitamin supplements, etc. During the face to face interview, physical exercise was evaluated via an 8-item standardized questionnaire. Average time spent on commonly leisure-time physical exercises (including trotting, running, climbing, ball game, swimming, gymnastic, yoga, etc.) in a typical week in past 1 year were reported by each participant. Height and weight were measured with subjects dressed in light clothes and shoes-off in standing position. Each measurement was taken twice and the average value was calculated for further analyses. Body mass index (BMI, in kg/m^2^) was then calculated.

#### The BMD and skeletal muscle mass measurement

We used dual-energy X-ray absorptiometry (DXA) (Hologic QDR-4500, Waltham, MA, USA) to determine the BMD forthe whole body, lumbar spine(L1-L4), and hip sites. The *in vivo* coefficients of variation (CV) of the BMD measurements were 0.87% (whole body), 1.02% (spine), 1.18% (total hip) and 1.92% (femoral neck), respectively. The long-term CV of the daily measurements of the spine phantom was 0.26% between April 2011 to September 2015. A whole body scan was used to quantify the body fat and appendicular skeletal muscle mass (ASM), using the same instrument. The coefficient variations (CV) of the muscle mass measurement were 2.6% for left arm, 3.2% for right arm, 4.3% for left leg and 4.0% for right leg, respectively. The ASM was defined as the sum of the lean mass of the arms and legs [37, 38]. The skeletal muscle mass index (SMI) of the whole body was calculated using the following formula, SMI = ASM/Height^2^(in kg/m^2^) [38]. According to the same formula, the arm and leg SMIs were also calculated [37].

#### Blood analysis

Overnight fasting blood was collected and the serum was separated within 2 hours and stored at -80°C until analyzed. The measurement of UA was performed using the enzymatic colorimetric method (Fenghui, Shanghai, China). All serum analyses were performed using a Hitachi 7600–010 automatic analyzer (Hitachi, Tokyo, Japan), and the variation coefficients was 5.46% for UA at baseline and the follow up.

### Statistical analysis

Participants were divided into gender-stratified quartiles [Q1 (lowest), Q2, Q3, and Q4] according to serum UA value. The continuous variables were expressed as the mean and standard deviation (SD). A comparison of the means across the quartiles was performed using a one-way ANOVA, or the Mann-Whitney nonparametric test in the case of non-normal distribution. The categorical variable was presented as a number or a percentage, and the Pearson chi-square test was used to examine the difference in frequency.

Analysis of covariance (ANCOVA) was used to examine the quartile differences in BMD and SMI. The multiplicative interactions between UA and gender were estimated by adding the interaction terms. As no significant interactions were found between the UA levels and gender (P for interactions: 0.153–0.860), all of the data analyses were then performed for men and women combined. In the ANCOVA models, we adjusted for age, gender, height, weight, blood pressure, education level, years since menopause, physical exercise, smoking, alcohol drinking, use of calcium and multivitamin supplements, medications affecting bone metabolism (including hormone therapy, bisphosphonate, calcitonin, vitamin D, *etc*.), chronic hepatitis, diabetes, and cardiovascular diseases. Multiple comparisons between quartile groups were conducted using the Bonferroni test. Path analysis, via structural equation modeling, including the variables serum UA, mediator (SMI), and BMDs at all sites, was performed to examine and quantify the proposed theoretical model, that is, the direct or the indirect effect via SMI of UA on BMD variance. To attenuate the short-term fluctuation of a single assessment of UA, we additionally examined in retrospect baseline serum UA among participants successfully followed up and utilized an average value of baseline and follow-up UA as exposure parameter, and further re-examined the relationship between UA and BMDs, SMI.

Data analyses were carried out using the SPSS for Windows statistical software package version 13.0 (SPSS Inc., Chicago, IL, USA), except for the path analysis, which was performed using AMOS 17.0. The two-sided value was considered statistically significant at p < 0.05.

## Results

### The characteristics of participants

Table 1 shows the participants’ characteristics, serum data, and appendicular skeletal muscle mass (ASM)for the 963 men and 2116 women across the UA quartiles. Individuals with higher UA levels had greater values of age, body weight, BMI, blood pressure, and YSM, but lower educational level. There were no significant differences in cigarette smoking, alcohol drinking, physical exercise, use of calcium and multivitamin supplements, and medication use between the quartiles. Means (SD) of UA value across quartiles were 257 (37), 318 (30), 366 (34), and 451 (60) μmol/L for Q1 to Q4 (Table 1).

**Table 1:** The characteristics of participants by quartiles of UA (n = 3079).

	Q1(n = 769)	Q2(n = 770)	Q3(n = 770)	Q4(n = 770)	P-Diff
Age (years)	57.5±5.9	57.6±6.4	58.0±6.2	58.9±6.3	***<0*.*001***
Weight (kg)	56.6±9.4	58.6±9.4	60.3±9.7	61.6±9.7	***<0*.*001***
Height (cm)	158.4±7.4	158.6±7.5	158.5±7.5	158.3±7.6	0.824
BMI	22.5±2.9	23.2±3.0	23.9±3.0	24.5±3.1	***<0*.*001***
SBP (mm Hg)	123±18	124±18	126±18	128±18	***<0*.*001***
DBP (mm Hg)	74±10	75±10	76±10	77±10	***<0*.*001***
Educational level (%)					**0.027**
Secondary school or below	6.6	6.4	7.2	8.3	
High school	12.1	12.1	11.3	11.0	
College or above	6.2	6.5	6.5	5.7	
YSM, y	6.8±5.6	7.5±6.5	7.9±6.5	8.5±6.7	***<0*.*001***
Smoker (%)	2.6	3.3	3.0	2.7	0.295
Alcohol drinker (%)	1.8	2.1	2.2	1.9	0.702
Physical exercise (%)					0.816
Never	7.7	8.2	8.1	8.4	
< 60 min per day	12.0	11.7	11.9	12.0	
≥60 min per day	5.3	5.0	5.1	4.5	
User of Ca supplement (%)	7.4	8.0	7.3	7.0	0.380
User of Vitamins(%)	4.4	5.4	4.5	4.4	0.168
Drug history ^a^ (%)	3.6	3.9	3.6	3.5	0.841
Serum UA (μmol/L)	257±37	318±30	366±34	451±60	
Leg lean mass (kg)	12.64±2.66	12.98±2.73	13.26±2.80	13.43±2.76	***<0*.*001***
Arm lean mass (kg)	4.04±1.09	4.12±1.15	4.19±1.15	4.24±1.15	**0.003**
ASM (kg)	16.68±3.69	17.10±3.82	17.45±3.89	17.67±3.86	***<0*.*001***

Abbreviations: UA, uric acid; BMI, body mass index; SBP, systolic blood pressure; DBP, diastolic blood pressure; ASM, appendicular skeleton muscle mass; YSM, years since menopause in women. Continuous variables were described by means ± SD. Categorical variables were presented as number (%).

^a^: including hormone therapy, bisphosphonate, calcitonin, vitamin D, etc.

**P-Diff.** p for overall difference across the quartiles.

### Covariate-adjusted BMD by the UA quartiles

There was a significantly dose-dependent and positive association between the serum UA and the BMDs at all sites after adjusting for age and gender in Model 1 (all p-trends <0.001). The mean BMDs were 3.0% (whole body), 6.8% (lumbar spine), 4.8% (total hip), and 4.9% (femoral neck) greater in Q4 compared to in Q1. After further adjustment for other potential confounding factors in Model 2, the significant association remained. The mean BMDs in Q4 were 2.3% (whole body), 4.1% (lumbar spine), 2.4% (total hip), and 2.0% (femoral neck) higher than those in Q1 (Table 2).When analyzed by an average value of baseline and follow-up UA, the positive and significant association of UA with BMDs remained stable (all p-trends <0.001, S1 Table).

**Table 2:** Covariate-adjusted BMD by the UA quartiles (mean ± SEM, n = 3079).

BMD(g/cm^2^)	Q1 (n = 769)	Q2 (n = 770)	Q3 (n = 770)	Q4 (n = 770)	%Diff	P-Diff	P-trend
**Model 1**							
Whole Body	1.083±0.004	1.089±0.004	1.101±0.004**	1.116±0.004***	3.0	***<0*.*001***	***<0*.*001***
Lumber	0.858±0.005	0.865±0.005	0.895±0.005***	0.916±0.005***	6.8	***<0*.*001***	***<0*.*001***
Total Hip	0.811±0.010	0.817±0.010	0.856±0.010**	0.850±0.010*	4.8	**0.001**	***<0*.*001***
Femoral Neck	0.674±0.004	0.675±0.004	0.698±0.004***	0.707±0.004***	4.9	***<0*.*001***	***<0*.*001***
**Model 2**							
Whole Body	1.086±0.003	1.090±0.003	1.099±0.003*	1.111±0.003***	2.3	***<0*.*001***	***<0*.*001***
Lumber	0.869±0.005	0.869±0.005	0.891±0.005*	0.905±0.005***	4.1	***<0*.*001***	***<0*.*001***
Total Hip	0.820±0.010	0.821±0.010	0.852±0.010	0.840±0.010	2.4	0.057	**0.043**
Femoral Neck	0.684±0.004	0.678±0.003	0.694±0.003	0.698±0.004*	2.0	***<0*.*001***	***<0*.*001***

**Model 1:** adjusting for age, gender; **Model 2:** adjusting for age, gender, height, weight, blood pressure, educational level, years since menopause, physical exercise, smoking, drinking, Ca supplement, vitamin supplement, medications, chronic hepatitis, diabetes and cardiovascular diseases. (ANCOVA). Abbreviations: **P-Diff.** p for overall difference across the quartiles; **%Diff:** percentage difference = (Q4 –Q1) / Q1 100%.

*, **,***: compared with Q1, *: p<0.05, **: p<0.01, ***: p<0.001 **(**Bonferroni test).

### Covariate-adjusted SMI by the UA quartiles

A similarly positive and linear association between serum UA and muscle mass was found, after adjusting for a variety of potential covariates (all p-trends < 0.001). The mean values for total SMI and arm and leg SMIs were 5.5%, 4.5%, and 5.9% greater in quartile 4 (vs. 1) for UA in Model 2, respectively. Further controlling for whole-body fat mass in Model 3 (Table 3), those subjects in quartile 4 (vs. 1) revealed a 2.7% (total), 2.5% (arm), 2.7% (leg) greater SMI. When replacing the single measurement of UA with an average value of baseline and follow-up, the observed favorable association was still significant (all p-trends < 0.001, S2 Table).

**Table 3:** Covariate-adjusted SMI by the UA quartiles (mean ± SEM, n = 3079).

SMI(kg/m^2^)	Q1 (n = 769)	Q2 (n = 770)	Q3 (n = 770)	Q4 (n = 770)	%Diff	P-Diff	P-trend
**Model 1**							
SMI	6.583±0.026	6.726±0.026**	6.878±0.026***	6.990±0.026***	6.2	***<0*.*001***	***<0*.*001***
Arm SMI	1.589±0.007	1.615±0.007*	1.647±0.007***	1.672±0.007***	5.2	***<0*.*001***	***<0*.*001***
Leg SMI	4.993±0.020	5.111±0.020***	5.231±0.020***	5.317±0.020***	6.5	***<0*.*001***	***<0*.*001***
**Model 2**							
SMI	6.603±0.026	6.736±0.025**	6.870±0.025***	6.967±0.026***	5.5	***<0*.*001***	***<0*.*001***
Arm SMI	1.594±0.007	1.618±0.007	1.645±0.007***	1.666±0.007***	4.5	***<0*.*001***	***<0*.*001***
Leg SMI	5.008±0.020	5.118±0.020**	5.225±0.020***	5.301±0.020***	5.9	***<0*.*001***	***<0*.*001***
**Model 3**							
SMI	6.698±0.023	6.766±0.023	6.834±0.023***	6.878±0.023***	2.7	***<0*.*001***	***<0*.*001***
Arm SMI	1.610±0.007	1.623±0.006	1.639±0.006*	1.651±0.007***	2.5	***<0*.*001***	***<0*.*001***
Leg SMI	5.088±0.018	5.142±0.018	5.195±0.018***	5.227±0.018***	2.7	***<0*.*001***	***<0*.*001***

**Model 1:** adjusting for age, gender; **Model 2:**adjusting for age, gender, blood pressure, educational level, years since menopause, physical exercise, smoking, drinking, Ca supplement, vitamin supplement, drug history, chronic hepatitis, diabetes and cardiovascular disease; **Model3:** Model 2 + whole-body fat mass. (ANCOVA). Abbreviations: **P-Diff.:** p for overall difference across the quartiles; **%Diff:** percentage difference = (Q4 –Q1) / Q1 ×100%; **SMI:**skeletal muscle mass index SMI = ASM/Height^2^**(**kg/m^2^**)**; **ASM:** appendicular skeleton muscle mass.

*, **,***: compared with Q1, *: p<0.05, **: p<0.01, ***:p<0.001 **(**Bonferroni test).

### The path analysis result

**Fig 1:**
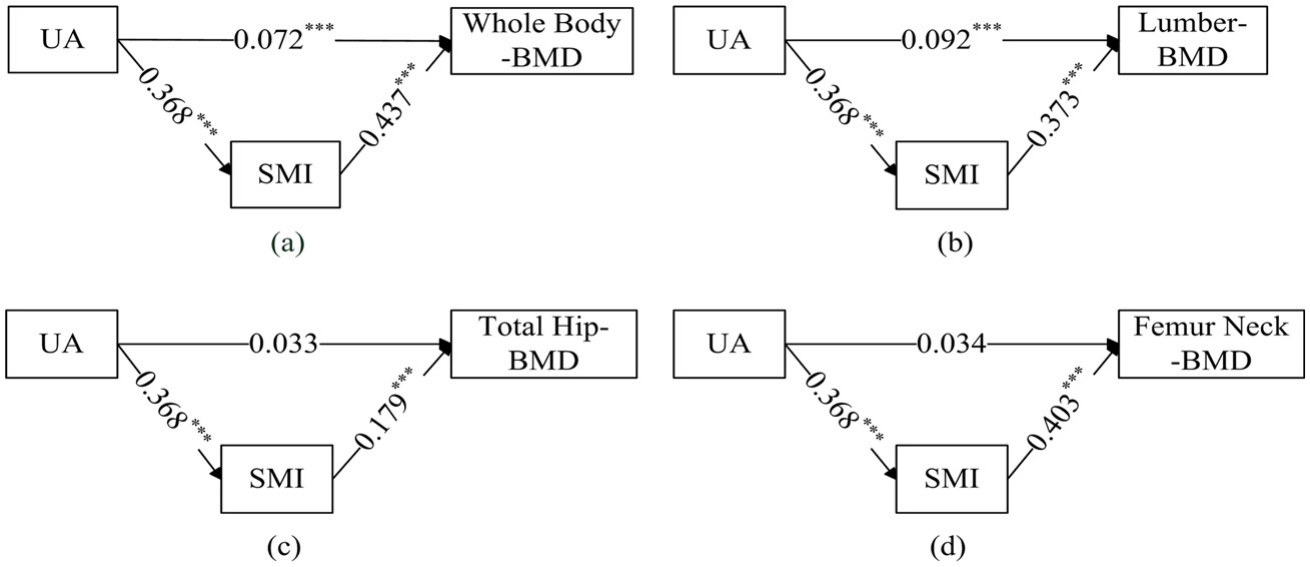
Path analyses of associations among serum UA, mediators (SMI), and BMD at whole body, lumber, total hip, femoral neck in Chinese middle-age and older adults (values are standardized regression weights, *: p<0.05, **: p<0.01, ***: p<0.001). Abbreviations: **UA**: uric acid; **SMI:** skeletal muscle mass index; **BMD:** bone mineral density.

At whole body and lumber, serum UA showed both directly and indirectly positive associations with the BMDs in the path analysis (all p < 0.001, Fig 1).The standardized regression weights for the direct association were 0.072 (whole-body), 0.092 (lumber). By contrast, the indirect association mediated by increasing SMI tended to be more pronounced. The standardized regression weights were 0.161 (whole-body), 0.137 (lumber). For total hip and femoral neck sides, UA only indicated an indirectly significant relationship by mediating SMI with the BMDs (standardized regression weights = 0.066, 0.149; both p < 0.001). A significantly direct association was not found between UA and BMDs at total hip or femoral neck.

## Discussion

In this community-based study pooling 3079 middle-aged and elderly Chinese adults, we found that higher serum UA was significantly and dose-dependently associated with a greater BMD and skeletal muscle mass indices, suggesting a potentially protective effect against musculoskeletal aging. In addition, path analysis revealed that the beneficial association between serum UA and the BMD might be partly contributed to an indirect pathway, mediated by increasing muscle mass.

### An association between UA and BMD

Previous epidemiological studies examined the relationship between serum UA and bone health. Results from cross-sectional analyses showed that serum UA was favorably associated with bone health (greater BMDs, lower prevalence of fracture or osteoporosis) among 1,705 older Australian men (70 years) [25], in 7,502 postmenopausal Korean women [26], in 615 peri- and postmenopausal Japanese women [27], in 5,074 participants of The Rotterdam Study [39], and in 17,735 Chinese adults (50 years) for health examination [28], but not in 6,759 National Health and Nutrition Examination Survey (NHANES; 2005–2010) participants (30 years) [29]. Longitudinal studies found that higher serum UA might attenuate bone loss among 356 peri- and post-menopausal Australian women [31] and reduced the risk of osteoporotic fractures in 16,078 Korean men (50 years) [30], in 5074 participants of the Rotterdam Study [39], and in a case-cohort of older men (387 nonspine fracture cases and 1,383 controls) enrolled in the Osteoporotic Fractures in Men (MrOS) study [40], but did not decrease the risk of osteoporotic fractures among 1,586 older Italian adults followed up 4 years [32], and among 2,729 American older women form the Cardiovascular Health Study (CHS) over ten years of follow-up [33]. Moreover, a U- shaped relationship between serum urate levels and hip fractures was observed in 1,963 men in the CHS [33].

In overall, our findings were consistent with the results from the majority of the previous studies, which supported the hypothesis that UA is beneficial to bone health in human. The between-study heterogeneity among previous results might be explained by the variations in population characteristics (e.g., ethics, gender, age, etc.), study design, study size, and covariates adjusted for. The favorable association appeared to be more frequently observed in Asians [26–28, 30] than in Westerns [29, 32, 33], in those with larger study size [30, 39], in cross-sectional (*vs*. longitudinal) studies, in those using indices of BMD [31] (*vs*. fractures [32]), and in the models with less number of adjusted covariates [29].

### An association between UA and skeletal muscle

A limited number of population studies have tested the relationship between UA and skeletal muscle, generating inconsistent results. Similar to our results, Beberashvili *et al*. reported that the lean body mass and the mid-arm muscle circumference increased with an increase in UA tertiles in 216 maintenance hemodialysis patients [41]. In addition, a positive link between UA and muscle/handgrip strength was also observed in a few small populations in cross-sectional studies [41–43]. However, a large cross-sectional study of 7,544 participants (NHANES III), revealed that the serum UA level was positively associated with an increased risk of sarcopenia (OR = 1.1, 95% CI:1.1–1.2), diagnosed by SMI (SMI = ASM/Weight × 100%) measured using bioelectrical impedance analysis (BIA) [44]. A possible explanation for the discrepancy might be due to the differences in the methods of SMI calculation and muscle mass measurement. The different definitions of SMI make it difficult to directly compare the study results. Besides, for the assessment of muscle mass, BIA tend to overestimate the muscle mass compared with the DXA and showed a low concordance with the DXA for the diagnosis of sarcopenia (*Kappa* = 0.48) [45]. More studies on muscle mass accompanying muscle performance were needed to address this issue.

### Mechanisms for favorable associations

A possible mechanism explaining the potentially beneficial effect of serum UA on bone and muscle might be related to its antioxidant capacity. UA, as a reactive water-soluble antioxidant, reacts with various oxidants and is thought to be responsible for over half amount of the antioxidant properties of plasma [21]. Accumulating evidence suggests that oxidative stress could play a critical role in bone loss and muscle aging [13, 46]. In aging cells, with an increase in ROS, oxidative stress not only causes direct damage to proteins, lipids, and DNA macromolecules, but also induces the activation and transfer into the nucleus of forkhead homeobox type O (FOXO) transcription factors, initiating a defense mechanism targeting oxidative stress [47]. Many lines of evidence suggest that the exacerbated activation of FOXOs is likely to inhibit osteoblast generation by disturbing the Wnt/β-catenin signaling pathway [48] and promoting myofibrillar proteolysis and muscle atrophy through the activation of the muscle atrophy F-box (MAFbx/atrogin-1) and the muscle RING-finger protein-1 (MuRF1) [49, 50]. *In vitro* experiments showed that UA treatment significantly decreases the ROS content of osteoclast precursors and osteoclastogenesis [26] and induces osteoblast differentiation [51]. Thus, serum UA can logically be deemed a protective factor for bone and muscle metabolism, via its ROS scavenging activity.

### UA-BMD association partly mediated by muscle mass

In this study, our findings showed that the beneficial UA-BMD association might be partly mediated by muscle mass. It is well established that greater muscle mass is associated with better bone health in population-based studies. A cross-sectional study showed a positive association between lean mass and BMD in 6,249 Italian women [52]. In the study by Cheng *et al*., lean mass had a significantly favorable association with BMDs at the total body, spine, and femur in 1,475 Chinese men and 1,534 women [53]. Mechanical loading, originating from the muscle, is translated to the bone by direct physical stimulation and is essential to bone maintenance during adulthood [3]. Moreover, previous studies showed that some muscle-derived cytokines (e.g., insulin-like growth factor 1 and myostatin) might also regulate bone metabolism via the paracrine and endocrine pathways, implying a molecular crosstalk between the muscle and bone [5]. Therefore, it is rational to hypothesize that UA exerts an indirect positive effect on bone maintenance by benefiting muscle metabolism, as mentioned above.

### Strengths and limitations

This study has several strengths. To the best of our knowledge, this is the first study to report a favorable association between UA and muscle mass and to show that the UA-BMD association is partly mediated by muscle mass. Secondly, among participants successfully followed up, we additionally retrospectively examined their baseline serum UA value, then calculated an average value of baseline and follow-up UA as exposure parameter and re-examined the relationship between UA, BMDs and SMI, attenuating the short-term fluctuation of a single assessment of UA. Thirdly, a relatively large study size of this present study allowed us to achieve more precise results.

The limitations of this study also deserve attention. First, the largest limitation is absence of muscle performance measurements, which hindered a further exploration of the relationship between UA, muscle performance and bone. Second, the cross-sectional study design did not allow us to infer a cause–effect relationship, although an average value of baseline and follow-up UA in supplemental data might represent a long-term exposure level and then to some extent reduce the possibility of reverse causality. Third, we measured the skeletal muscle mass by the DXA technique rather than the golden methods by MRI or CT. However, previous studies have showed an excellent validity between results determined by DXA as compared to those by MRI and CT (*R*^2^ = 0.96) [54, 55]. Last, we did not measure other antioxidants and antioxidant enzymes in plasma that might contribute to a residual confounding effect, although we adjusted for many other potentially confounding factors.

### Clinical significances

UA has long been known for its harmful effects such as gouts and uric lithiasis, as well as its potential association with hypertension, metabolism syndrome, renal disease and cardiovascular diseases. A large number of studies have been focusing on the clinical significance of lowering circulating UA to prevent/improve the chronic diseases via diet or relevent medications [56–58]. However, many previous studies suggested that UA might be benefit to the maintenance of bone health and the prevention of some degenerative diseases (i.e., neurological diseases and cancers) [22–24]. In this study, we repeatedly found the favorable UA-BMD association as did in the majority of the previous studies, and firstly reported that higher levels of serum UA (studies mean range: 257–451 μmol/L) were associated with greater levels of skeletal muscle mass. Our findings, together with previous results, suggested that clinicians might need to weigh the pros and cons of UA reduction, especially in aging populations with rapid bone and muscle loss, or degenerative disease of the nervous system. Considering the null effects of raising serum uric acid on BMD and bone quality in rats [29], further interventional studies (i.e., treatments against high UA) are needed to verify whether there is a causal relationship between UA and bone/muscle health in humans.

## Conclusions

In summary, our study revealed that the serum UA level was positively and significantly associated with both the BMD and the skeletal muscle mass, and suggested that the favorable UA-BMD association might be partly mediated by skeletal muscle mass in Chinese middle-aged and elderly adults. Our findings support the hypothesis that UA exerts a beneficial effect on bone and muscle metabolism. Due to limited experimental evidence available, further interventional studies (particularly in humans) are needed to clarify if the favorable associations are causal or not.

## Supporting Information

S1 Table Covariate-adjusted BMDs by quartiles of an average value of UA at baseline and follow-up (mean ± SEM, n = 2355). (DOCX)

S2 Table Covariate-adjusted SMI by quartiles of an average value of UA at baseline and follow-up (mean ± SEM, n = 2355). (DOCX)
