# Supplementary material for: Elevated Serum Uric Acid Is Associated with Greater Bone Mineral Density and Skeletal Muscle Mass in Middle-Aged and Older Adults
Source: PLoS One. 2016 May 4;11(5):e0154692. doi: 10.1371/journal.pone.0154692 (PMC4856375; doi:10.1371/journal.pone.0154692)
Supplement: S1 Table — (DOCX) [file pone.0154692.s001.docx]

**S1 Table. Covariate-adjusted BMDs by quarters of an average value of UA at baseline and follow-up** (mean ± SEM, n=2355)

| **BMD**(g/cm^2^) | **Q1** (n=588) | **Q2** (n=589) | **Q3** (n=589) | **Q4** (n=589) | **%Diff** | **P-Diff** | **P-trend** |
| --- | --- | --- | --- | --- | --- | --- | --- |
| **Model 1** | | | | | | | |
| Whole Body | 1.081±0.004 | 1.088±0.004 | 1.094±0.004 | 1.115±0.004**^***^** | 3.1 | ***<0.001*** | ***<0.001*** |
| Lumber | 0.851±0.006 | 0.870±0.006 | 0.881±0.006**^**^** | 0.918±0.006**^***^** | 7.9 | ***<0.001*** | ***<0.001*** |
| Total Hip | 0.806±0.004 | 0.828±0.004**^**^** | 0.833±0.004**^***^** | 0.861±0.004**^***^** | 6.8 | ***<0.001*** | ***<0.001*** |
| Femoral Neck | 0.671±0.004 | 0.681±0.004 | 0.690±0.004**^**^** | 0.714±0.004**^***^** | 6.4 | ***<0.001*** | ***<0.001*** |
| **Model 2** | | | | | | | |
| Whole Body | 1.085±0.004 | 1.089±0.004 | 1.094±0.004 | 1.110±0.004**^***^** | 2.3 | ***<0.001*** | ***<0.001*** |
| Lumber | 0.862±0.006 | 0.874±0.005 | 0.880±0.005 | 0.904±0.006**^***^** | 4.9 | ***<0.001*** | ***<0.001*** |
| Total Hip | 0.817±0.004 | 0.832±0.004**^*^** | 0.832±0.004 | 0.847±0.004**^***^** | 3.7 | ***<0.001*** | ***<0.001*** |
| Femoral Neck | 0.681±0.004 | 0.684±0.004 | 0.689±0.004 | 0.702±0.004**^**^** | 3.1 | **0.001** | ***<0.001*** |

**Model 1:** adjusting for age, gender; **Model 2:** adjusting for age, gender, height, weight, blood pressure, educational level, years since menopause, physical exercise, smoking, drinking, Ca supplement, vitamin supplement, drug history, chronic hepatitis, diabetes and cardiovascular disease. (ANCOVA)

**P-Diff.:** p for overall difference across the quartiles.

**%Diff:** percentage difference = (Q4 – Q1) / Q1 ×100%.

***, **,***:** compared with Q1, ***:** p<0.05, ****:** p<0.01**, ***:** p<0.001 **(**Bonferroni test) .
