# Supplementary material for: Elevated Serum Uric Acid Is Associated with Greater Bone Mineral Density and Skeletal Muscle Mass in Middle-Aged and Older Adults
Source: PLoS One. 2016 May 4;11(5):e0154692. doi: 10.1371/journal.pone.0154692 (PMC4856375; doi:10.1371/journal.pone.0154692)
Supplement: S2 Table — (DOCX) [file pone.0154692.s002.docx]

**S2 Table.** **Covariate-adjusted SMI by quarters of an average value of UA at baseline and follow-up** (mean ± SEM, n=2355)

| **SMI** (kg/m^2^) | **Q1** (n=588) | **Q2** (n=589) | **Q3** (n=589) | **Q4** (n=589) | **%Diff** | **P-Diff** | **P-trend** |
| --- | --- | --- | --- | --- | --- | --- | --- |
| **Model 1** | | | | | | | |
| SMI | 6.512±0.030 | 6.639±0.030**^*^** | 6.739±0.030**^***^** | 6.943±0.030**^***^** | 6.6 | ***<0.001*** | ***<0.001*** |
| Arm SMI | 1.570±0.008 | 1.593±0.008 | 1.615±0.008**^***^** | 1.664±0.008**^***^** | 6.0 | ***<0.001*** | ***<0.001*** |
| Leg SMI | 4.942±0.023 | 5.046±0.023**^*^** | 5.124±0.023**^***^** | 5.279±0.023**^***^** | 6.8 | ***<0.001*** | ***<0.001*** |
| **Model 2** | | | | | | | |
| SMI | 6.532±0.029 | 6.652±0.029**^*^** | 6.728±0.029**^***^** | 6.919±0.029**^***^** | 5.9 | ***<0.001*** | ***<0.001*** |
| Arm SMI | 1.575±0.008 | 1.598±0.008 | 1.613±0.008**^**^** | 1.657±0.008**^***^** | 5.2 | ***<0.001*** | ***<0.001*** |
| Leg SMI | 4.958±0.023 | 5.055±0.023**^*^** | 5.115±0.023**^***^** | 5.262±0.023**^***^** | 6.1 | ***<0.001*** | ***<0.001*** |
| **Model 3** | | | | | | | |
| SMI | 6.629±0.026 | 6.684±0.026 | 6.714±0.026 | 6.805±0.026**^***^** | 2.7 | ***<0.001*** | ***<0.001*** |
| Arm SMI | 1.590±0.007 | 1.603±0.007 | 1.610±0.007 | 1.639±0.007**^***^** | 3.1 | ***<0.001*** | ***<0.001*** |
| Le Leg SMI | 5.039±0.020 | 5.081±0.020 | 5.104±0.020 | 5.167±0.020**^***^** | 2.5 | ***<0.001*** | ***<0.001*** |

**Model 1:** adjusting for age, gender; **Model 2:** adjusting for age, gender, blood pressure, educational level, years since menopause, physical exercise, smoking, drinking, Ca supplement, vitamin supplement, drug history, chronic hepatitis, diabetes and cardiovascular disease; **Model 3:** Model 2 + whole-body fat mass. (ANCOVA)

**P-Diff.:** p for overall difference across the quartiles.

**%Diff:** percentage difference = (Q4 – Q1) / Q1 ×100%, ***, **,***:** compared with Q1, ***:** p<0.05, ****:** p<0.01**, ***:** p<0.001 **(**Bonferroni test); **SMI:** skeletal muscle mass index SMI = ASM /height^2^ **(kg/m^2^)**, **ASM:** appendicular skeleton muscle mass.
